# The effects of transfection reagent polyethyleneimine (PEI) and non-targeting control siRNAs on global gene expression in human aortic smooth muscle cells

**DOI:** 10.1186/s12864-015-2267-9

**Published:** 2016-01-05

**Authors:** Nurazhani A. Raof, Deepa Rajamani, Hsun-Chieh Chu, Aniket Gurav, Joel M. Johnson, Frank W. LoGerfo, Leena Pradhan-Nabzdyk, Manoj Bhasin

**Affiliations:** The Frank W. LoGerfo Division of Vascular and Endovascular Surgery, Beth Israel Deaconess Medical Center, Harvard Medical School, 330 Brookline Ave, Stoneman 8 M-10E, Boston, 02215 MA USA; Division of Interdisciplinary Medicine and Biotechnology, Genomics and Proteomics Center, Beth Israel Deaconess Medical Center, Harvard Medical School, 99 Brookline Avenue, Boston, MA 02215 USA; Department of Medicine, National Yang-Ming University, School of Medicine, Taipei City, Taiwan

**Keywords:** RNAi, Transfection reagent, PEI, Control siRNA

## Abstract

**Background:**

RNA interference (RNAi) is a powerful platform utilized to target transcription of specific genes and downregulate the protein product. To achieve effective silencing, RNAi is usually applied to cells or tissue with a transfection reagent to enhance entry into cells. A commonly used control is the same transfection reagent plus a “noncoding RNAi”. However, this does not control for the genomic response to the transfection reagent alone or in combination with the noncoding RNAi. These control effects while not directly targeting the gene in question may influence expression of other genes that in turn alter expression of the target. The current study was prompted by our work focused on prevention of vascular bypass graft failure and our experience with gene silencing in human aortic smooth muscle cells (HAoSMCs) where we suspected that off target effects through this mechanism might be substantial. We have used Next Generation Sequencing (NGS) technology and bioinformatics analysis to examine the genomic response of HAoSMCs to the transfection reagent alone (polyethyleneimine (PEI)) or in combination with commercially obtained control small interfering RNA (siRNAs) (Dharmacon and Invitrogen).

**Results:**

Compared to untreated cells, global gene expression of HAoSMcs after transfection either with PEI or in combination with control siRNAs displayed significant alterations in gene transcriptome after 24 h. HAoSMCs transfected by PEI alone revealed alterations of 213 genes mainly involved in inflammatory and immune responses. HAoSMCs transfected by PEI complexed with siRNA from either Dharmacon or Invitrogen showed substantial gene variation of 113 and 85 genes respectively. Transfection of cells with only PEI or with PEI and control siRNAs resulted in identification of 20 set of overlapping altered genes. Further, systems biology analysis revealed key master regulators in cells transfected with control siRNAs including the cytokine, Interleukin (IL)-1, transcription factor GATA Binding Protein (GATA)-4 and the methylation enzyme, Enhancer of zeste homolog 2 (EZH-2) a cytokine with an apical role in initiating the inflammatory response.

**Conclusions:**

Significant off-target effects in HAoSMCs transfected with PEI alone or in combination with control siRNAs may lead to misleading conclusions concerning the effectiveness of a targeted siRNA strategy. The lack of structural information about transfection reagents and “non coding” siRNA is a hindrance in the development of siRNA based therapeutics.

**Electronic supplementary material:**

The online version of this article (doi:10.1186/s12864-015-2267-9) contains supplementary material, which is available to authorized users.

## Background

RNAi is an emerging technology using a natural mechanism to inhibit gene expression via the degradation of target mRNAs through siRNA. We have been exploring RNAi for the purpose of modifying the vascular response to injury especially during graft implantation, employing endothelial cells and vascular smooth muscle cells, under various conditions and technology for siRNA delivery [1–4]. Although these studies have displayed promise of RNAi therapy as an alternative means to treat vascular diseases, there is so far no study discussing how transfection reagent alone and in combination with non-targeting (control) siRNAs affect gene expression level.

Our group is interested in developing siRNA based therapies to treat vascular bypass grafts, both vein and prosthetic, to prevent vascular graft failure. Injury to the graft and the host artery during graft implantation is the most important trigger for the downstream graft failure. Vascular smooth muscle cells play a major role in the failure process and thus are often targeted for therapeutic intervention. Thus in the present study the cells chosen were HAoSMCs [1–4].

When determining the effectiveness of siRNA silencing of a target gene, the standard control has been a comparison with control siRNA. In our laboratory we have used a series of controls. For example, if using a transfection reagent, our controls would be: 1. Saline alone. 2. Transfection reagent alone. 3. Transfection reagent plus control siRNA. We have noticed differences between these controls with regard to expression of the target gene, raising the question of the control effects on total genomic expression. For example, if the transfection reagent alters target gene expression and that is different from the transfection reagent plus the control siRNA, which is the better control? Can we count on the control siRNA to have no effect on target gene expression? Should saline or balanced salt solution be the appropriate control as it is least likely to affect target gene transcription? In this study, experiments were designed to determine the global effects on gene transcription of saline, a transfection reagent, and the transfection reagent plus control siRNA using RNA sequencing. Since our goal is to treat vascular bypass grafts with siRNA, we chose a transfection reagent PEI, as this is frequently used for in vivo applicability. Control siRNAs were obtained from Invitrogen and Dharmacon.

## Results

### Unsupervised clustering suggests that untreated (NT) and HAoSMCs treated with P, PI and PD are transcriptionally different

**Fig. 1:**
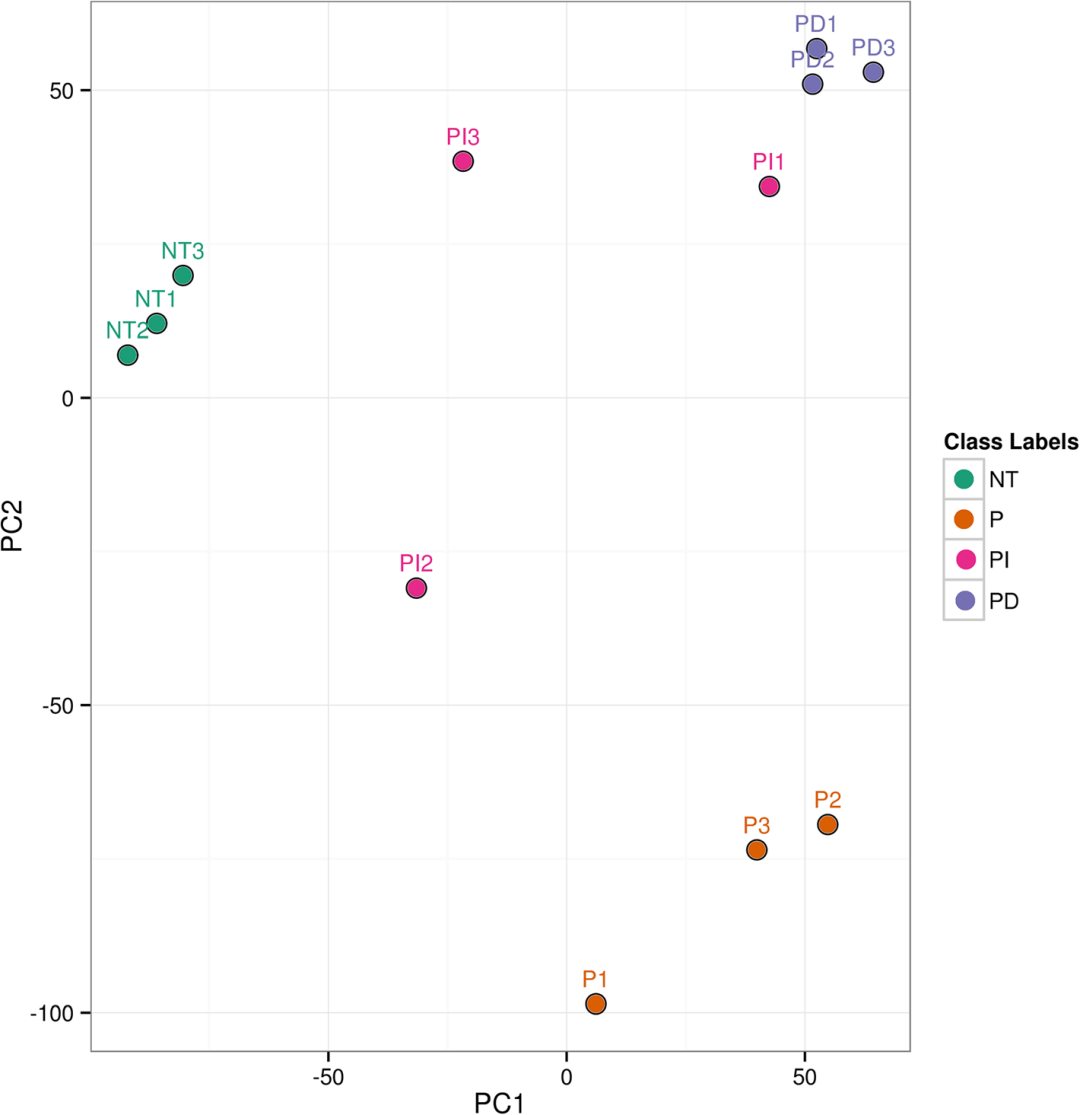
Principle Component Analysis (PCA) of HAoSMCs that were treated as follows: No Treatment (NT), PEI alone (P), PEI combined with control siRNA from Invitrogen (PI) and PEI combined with control siRNA from Dharmacon siRNA (PD). PCA analysis of three replicates in each treatment group suggests that NT and HAoSMCs treated with P, PI and PD form different clusters indicating that these treatment groups are transcriptionally different from each other

HAoSMCs were either treated with PEI alone (P), Invitrogen control siRNA complexed with PEI (PI) or Dharmacon control siRNA complexed with PEI (PD) for 24 h. HAoSMCs that were left untreated (NT) served as the experimental control. After treatment, cells were subjected to RNA isolation as described above. After preprocessing and normalization of RNA sequencing data we performed unsupervised analysis using PCA to determine relationship between different treatment groups as well as samples within each group. The unsupervised analysis demonstrated that samples are separated on the basis of transfection that is NT versus P, PI and PD along primary component (PC) 1 (Fig. 1). The samples from PEI alone group depicted maximum transcriptional differences as compared to control NT, PI and PD groups along primary component (PC) 2. Biological replicates from most of the groups clustered together indicating similar transcriptome profile.

### Inflammation and apoptosis related genes are upregulated/activated due to PEI transfection

**Fig. 2:**
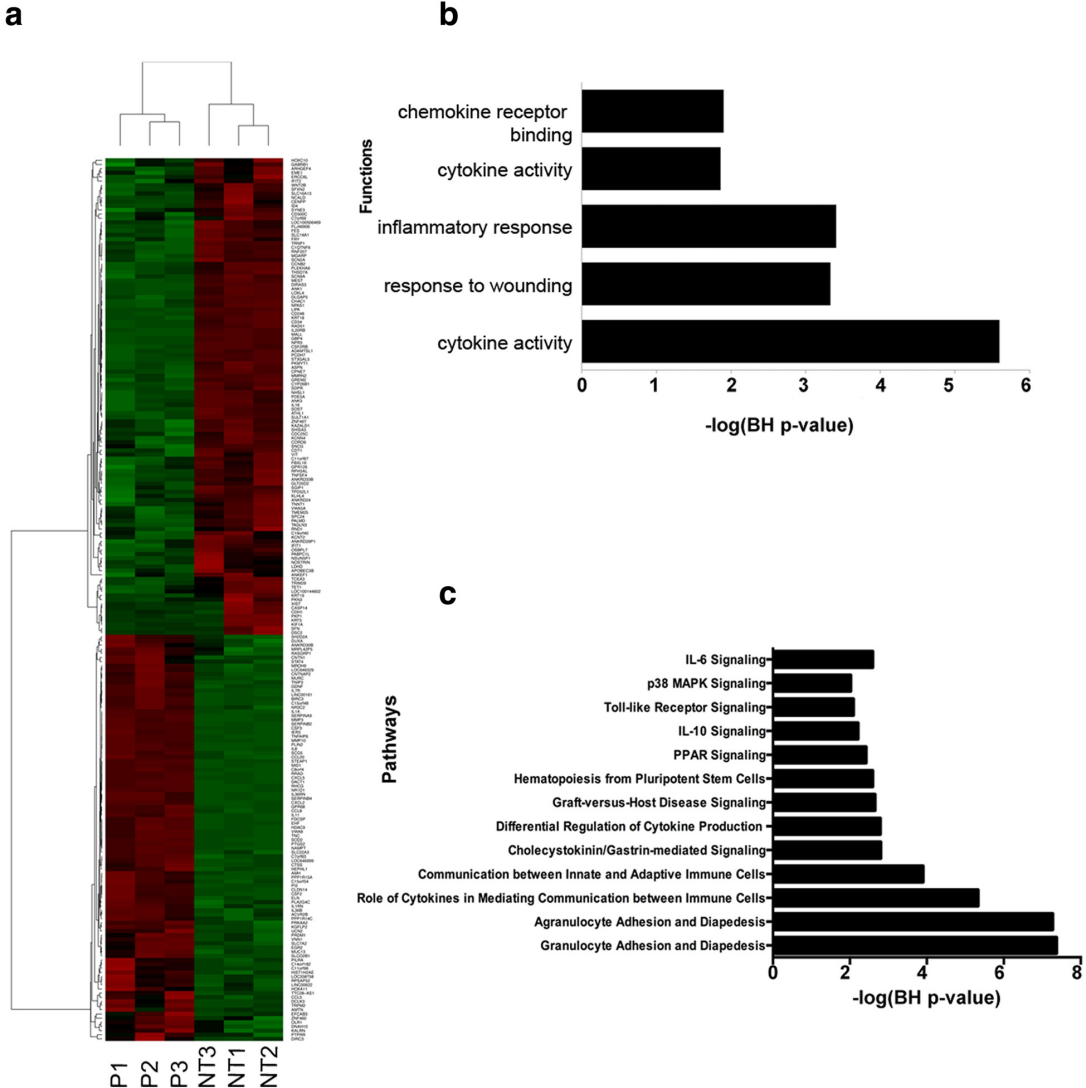
Transcriptional and biological characterization of alteration in HAoSMC induced by PEI. (**a**) Heatmap of genes that are significantly differentially expressed due to treatment with PEI alone (P) compared to No Treatment (NT). In the heatmap, rows depict differentially expressed genes and columns depict three replicates each of NT and P treated HAoSMC. The relative expression level of genes is shown using a pseudocolor scale from −3 to +3. Colors indicate standardized values (green represents down regulation and red represents up regulation). (**b**) Functional categories enrichment analysis of all significantly differentially expressed genes, and (**c**) Pathways enrichment analysis of all significantly differentially expressed genes

PEI (P), which comes in two forms; linear and branched polymer, has been extensively used as a non-viral cationic carrier to deliver drugs or genes into the cells via proton sponge effects [5]. The supervised comparison of NT samples with P only samples identified 213 significantly differentially expressed genes with false discovery rate <5 % and at least 2-fold change. Out of 213 differentially expressed genes, 115 and 98 were significantly downregulated and upregulated respectively (Fig. 2a and Additional file 1: Table S2). Treatment with P upregulated multiple genes linked to cell-mediated immune response and inflammatory response including Prostaglandin-Endoperoxide Synthase (PTGS) 2, Nicotinamide phosphoribosyltransferase (NAMPT), most prominently several chemokines and chemokine receptors genes such as Chemokine (C-X-C motif) Ligand (CXCL)-2, CXCL-11, CXCL-8, IL-1A, IL-11, Chemokine (C-C motif) Ligand (CCL)-5 and Colony Stimulating Factor (CSF)-2 (Fig. 2a).

To understand the underlying biological mechanism of alterations induced due to PEI treatment, we performed gene-ontology (GO) categories and canonical pathways analysis. The GO analysis of differentially expressed genes identified significantly affected categories (*P* value <0.01) that include cytokine activity, inflammatory response, chemokine activity and immune response (Fig. 2b). Furthermore, pathways analysis on the list of differentially expressed genes identified significant pathways (multiple test corrected *P* value <0.01) that include, Granulocyte Adhesion and Diapedesis, peroxisome proliferator-activated receptor (PPAR) signaling, IL-10 signaling, and IL-6 signaling (Fig. 2c). These pathways that are triggered by immune systems in response to PEI transfection, play a critical role in multiple diseases including cancer, immunological and neurodegenerative diseases. The activation of immune and inflammatory pathways 24 h after in vitro transfection with PEI at the vendor recommended N/P ratio in HAoSMCs suggests toxicity associated with PEI.

### Transcriptional alterations due to control siRNA compared to PEI alone

**Fig. 3:**
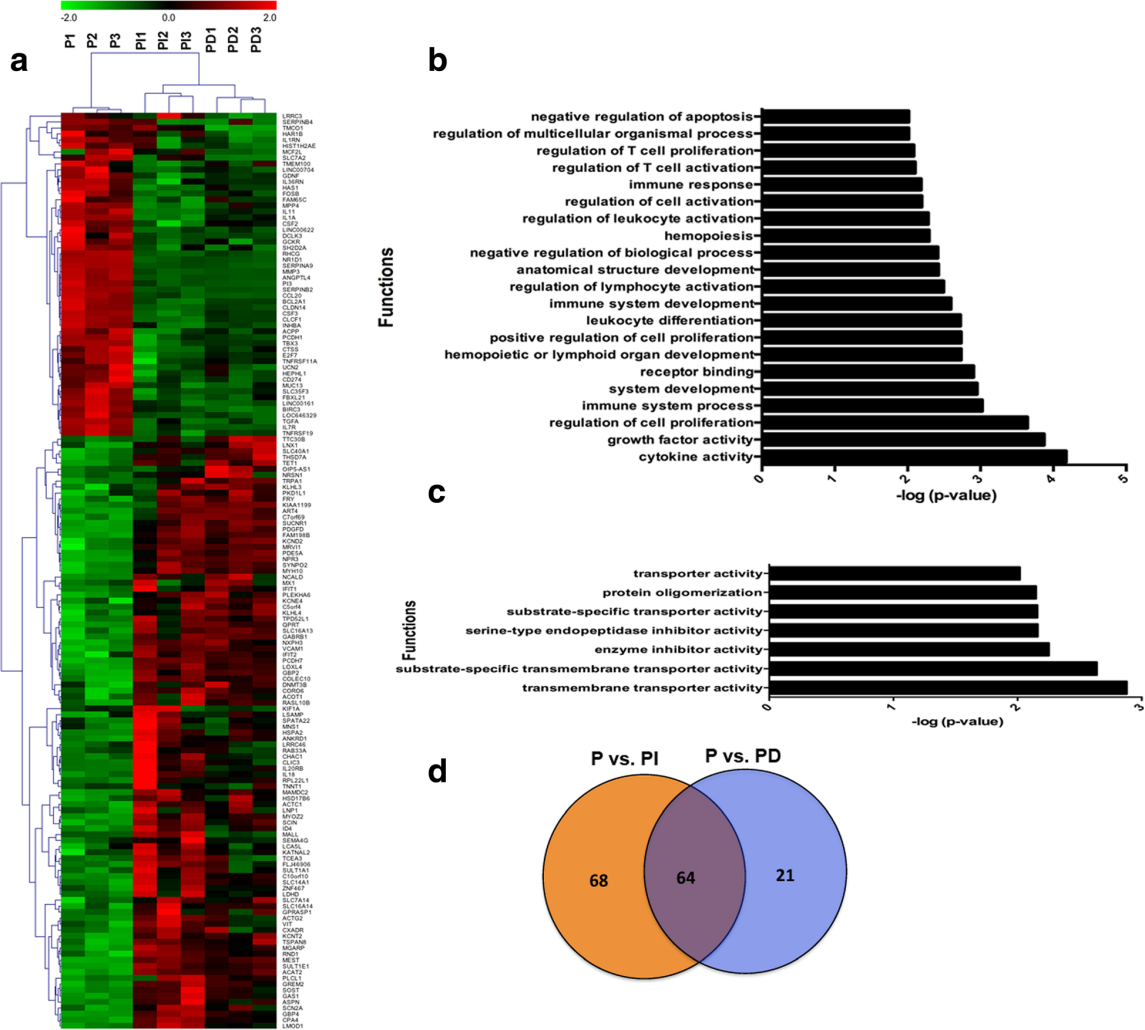
Transcriptional characterization of alteration induced in HAoSMC by transfection with PEI combined with control siRNA from Invitrogen (PI) or Dharmacon (PD). (**a**) Heat map of significantly differentially expressed genes due to transfection with PI or PD, (**b**) Functional categories significantly altered due to transfection with PI, (**c**) Functional categories that depict pattern of alteration (Raw *P* value < .01) due to transfection with PD. No functional category was found significant after multiple test correction, and (**d**) Venn diagram depicting genes common between HAoSMC treated with PI and PD

To understand the non-specific transcriptional alterations induced by control siRNA when combined with PEI, we performed global RNA sequencing on samples transfected with control unlabeled siRNA from Invitrogen or Dharmacon combined with PEI. The transfection of samples with control unlabeled siRNA from Invitrogen or Dharmacon compared to PEI alone significantly altered (FDR <5 % and FC > ± 2) 132 and 85 genes respectively (Fig. 3a and Additional file 1: Table S3A, S3B). A significant number (64) of genes were commonly altered by unlabeled siRNA from Invitrogen or Dharmacon (Fig. 3b and Additional file 1: Table S4) suggesting similar mechanisms that may affect transcriptional profile of the cells. To understand the biological mechanism underlying the alterations induced by control siRNA transfection, we performed functional and pathways enrichment analysis on the genes that were commonly altered by both Invitrogen and Dharmacon control siRNA. The functions uniting commonly differentially expressed genes were dominated by functions involved in cell proliferation and growth and immune/inflammatory response (Fig. 3c). Further pathways analysis on commonly affected genes depicted significant enrichment in inflammatory response pathways including Granulocyte/Agranulocyte Adhesion and Diapedesis. These pathways are the primary line of host defense against infection by bacterial pathogens and are rapidly recruited to sites of bacterial invasion suggesting that control siRNAs are probably recognized as foreign entities [6].

### Control siRNA and PEI lead to immune systems and inflammation related transcriptional changes

**Fig. 4:**
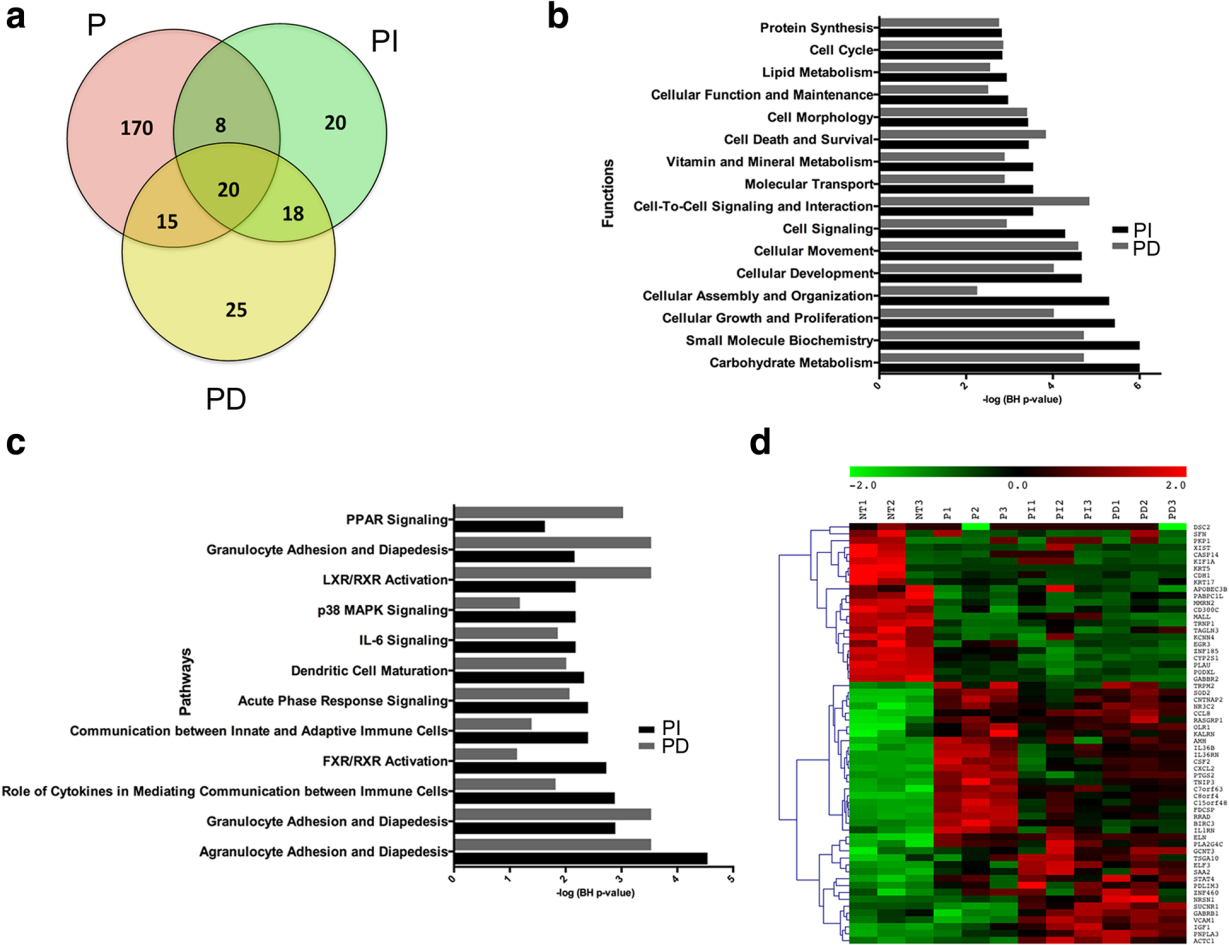
Transcriptional characterization of alteration induced in HAoSMC by PEI alone (P) or PEI combined with control siRNA from Invitrogen (PI) or Dharmacon (PD). (**a**) Venn Diagram depicting overlap among the genes that are differentially expressed in HAoSMC due to P alone, PI or PD as compared to no treatment (NT), (**b**) Functions significantly altered due to transfection with PI and PD, (**c**) Pathways significantly altered due to transfection with PI and PD, and (**d**) Heatmap of 20 common significantly differentially expressed genes due to transfection with P, PI or PD

Compared to NT, transfection of cells with either PI or PD resulted in significant transcriptional changes (Fig. 4a). The transfection of cells with PI resulted in dysregulation of 66 genes (Additional file 1: Table S5A), mostly dominated by genes linked to inflammation related pathways including “Agranulocyte/Granulocyte Adhesion and Diapedesis”, “Farnesoid X Receptor/ Retinoid X Receptor (FXR/RXR) Activation”, “Acute Phase Response Signaling”, “Dendritic Cell Maturation”, “IL-6 Signaling”, “p38 Mitogen-Activated Protein Kinase (MAPK) Signaling”, and “PPAR Signaling” (Fig. 4b). Similarly, transfection of cells with PD resulted in dysregulation of 78 genes (Additional file 1: Table S5B), which are dominated by genes linked to inflammation related pathways comparable to PI (Fig. 4b). Thus, transfection with both Dharmacon and Invitrogen non-targeting control siRNAs resulted in inflammation response similar to host defense response against infection of bacteria’s or viruses described above. The analysis identified a core set of 20 overlapping genes that are significantly altered due to transfection of cells with P, PI or PD (Table 1). This core set of overlapping genes is dominated by genes linked to immune/inflammatory and cell proliferation including Kinesin Family Member (KIF)-1A, STAT-4, CCL-8, Superoxide Dismutase (SOD)-2, IL-36B, CSF-2, and IL3-6RN (Fig. 4c). These genes have also been linked to vascular dysregulation and the associations are listed in Table 2.

**Table 1:** List of commonly significantly differentially expressed genes due to transfection of PEI alone (P), and PEI with Invitrogen (PI) and Dharmacon (PD) control siRNA respectively

Genes	P vs. NT			PI vs. NT			PD vs. NT		
	Log FC	*P* value	FDR	log FC	*P* value	FDR	Log FC	PV value	FDR
KRT5	−9.00108542	1.82E-08	9.11E-07	−9.00108542	1.12E-08	1.85E-06	−9.00108542	4.00E-09	5.77E-07
DSC2	−5.547567862	9.33E-06	0.000210938	−7.514505361	3.54E-07	3.79E-05	−5.843576549	2.01E-06	0.00010804
CASP14	−4.866920217	1.36E-06	3.98E-05	−6.718742357	9.08E-09	1.56E-06	−3.84210716	2.16E-05	0.000762738
PKP1	−4.065832231	4.03E-05	0.000734415	−5.837618386	4.72E-07	4.98E-05	−4.812633855	2.41E-06	0.000126389
SFN	−3.866190605	1.96E-05	0.000390234	−2.706646929	0.000813618	0.022294014	−5.116141429	1.68E-07	1.47E-05
XIST	−2.6415241	0.000447384	0.005355807	−4.189237025	9.64E-07	9.24E-05	−2.564333239	0.000399303	0.007636661
KIF1A	−2.37198378	0.001162629	0.011613329	−6.165808144	1.86E-09	3.89E-07	−2.639316704	0.000279027	0.005863775
PABPC1L	−1.469888511	5.36E-08	2.38E-06	−1.136168153	1.40E-05	0.000867902	−1.006563861	7.85E-05	0.002217316
MMRN2	−1.148174676	2.51E-05	0.000484772	−1.125962103	2.61E-05	0.00144306	−1.140277865	1.12E-05	0.00044101
STAT4	1.030820203	0.00090642	0.009359621	1.164708993	0.000135637	0.005691294	1.401188438	2.28E-06	0.000120413
ELN	1.327728145	5.88E-23	1.68E-20	1.389401412	3.20E-25	1.34E-21	1.303860319	1.21E-22	1.52E-19
CCL8	1.434021582	2.81E-11	2.43E-09	1.621652693	1.26E-14	8.32E-12	1.716686958	7.40E-17	3.37E-14
SOD2	1.461039459	3.51E-39	2.20E-36	1.007297504	7.52E-20	1.18E-16	1.329823799	5.71E-33	1.79E-29
CNTNAP2	1.550048726	6.82E-17	1.17E-14	1.167033839	4.31E-10	1.13E-07	1.302670483	1.60E-12	4.10E-10
C8orf4	1.657269124	1.89E-46	2.16E-43	1.214785377	1.07E-25	6.74E-22	1.229534359	1.34E-26	3.37E-23
NR3C2	1.839357043	6.15E-12	6.08E-10	1.505931617	3.47E-08	4.93E-06	1.89042933	2.94E-13	7.86E-11
IL36B	2.065054755	1.81E-13	2.09E-11	1.371901177	1.91E-06	0.000158997	1.623123119	7.63E-09	1.01E-06
CSF2	2.157136236	2.41E-36	1.38E-33	1.04449791	5.99E-10	1.46E-07	1.692407468	1.62E-23	2.77E-20
AMH	2.487063814	2.12E-08	1.05E-06	1.631133689	0.00045514	0.014311243	2.135611981	1.50E-06	8.61E-05
IL36RN	4.508694239	2.15E-34	1.12E-31	3.347303687	2.41E-18	2.33E-15	3.737917275	1.76E-23	2.77E-20

**Table 2:** Effects of the altered genes after transfection of cells with PEI and control siRNAs on vascular system in the literature

Genes	Effects on vascular system	References
KRT5	Overexpression enhanced vascular proliferation leading to cell hyperplasia in urinary bladder	[31]
DSC2	Mutations may predict arrhythmic events and sudden cardiac death	[32]
CASP14	Deletion may be the cause of vascular neurodegenerative disorder	[33]
PKP1	Overexpression in head and neck tumor tissues	[34]
SFN	Inhibited intimal hyperplasia in rat carotid injury model	[35]
MMRN2	Overexpression impaired tumor growth through VEGF pathway interference	[36]
STAT4	Upregulated during intimal hyperplasia	[9]
ELN	Aberrations caused aortic aneurysm	[37]
CCL8	Reduced macrophage accumulation in the vascular wall and blood pressure in hypertension.	[8]
SOD2	Protected against oxidative stress and endothelial dysfunction in carotid artery	[38]
C8orf4	Used as an endothelial inflammatory marker	[10]
NR3C2	Under expression may cause angiogenesis in colorectal carcinomas [39]	[39]
	Contributed to vascular remodeling and target organ damage [40]	[40]
CSF2	Stimulated arteriogenesis in pig peripheral artery disease model	[41]
AMH	Upregulation associated with varicose vein disease	[42]

### Systems biology oriented analysis of control siRNA transcriptional changes to identify key master regulators

**Fig. 5:**
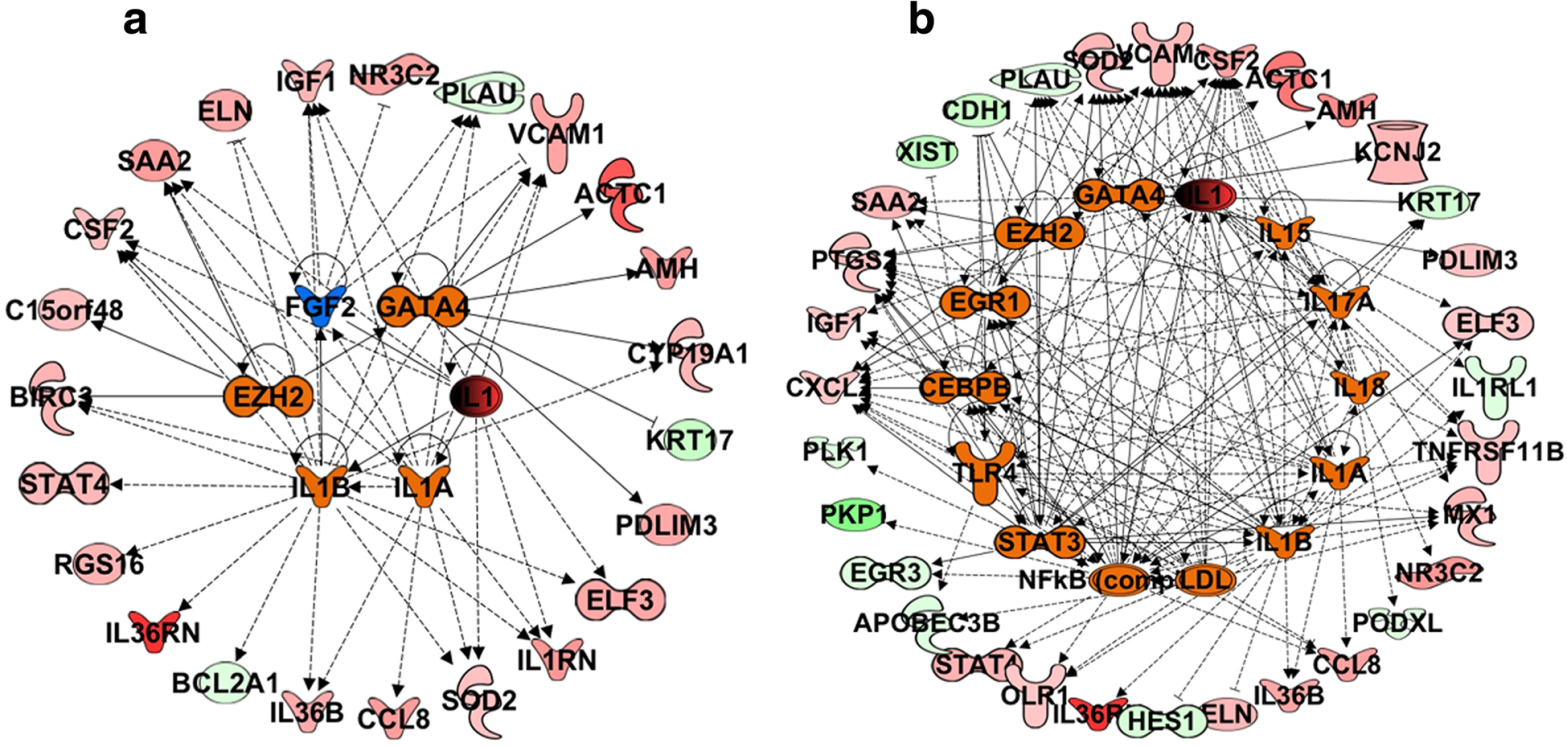
Interactive network of top regulatory molecules significantly altered in HAoSMC by treatment with PEI combined with control siRNA from Invitrogen (PI) or Dharmacon (PD). **a** PI, and (**b**) PD

To gain further insight into the impact of control siRNA transfection of cells, we performed systems biology oriented analysis on the transcripts that are differentially expressed after transfection with PI or PD. The regulatory analysis was performed using Ingenuity Pathway Analysis (IPA) 8.0 (www.ingenuity.com). The regulatory analysis of the transcripts that were differentially expressed after transfection with PI depicted activation of IL-1 response (Fig. 5a). IL-1 is a key inflammatory cytokine and its overexpression leads to inflammatory responses by initiating Nuclear Factor Kappa Beta (NFKB) cascade, edema, and the growth of leukocytes [7]. In addition to IL1, there was also a strong activation of immune response as illustrated by the upregulation of multiple proinflammatory chemokines and cytokines as well as NFKB cascade (e.g. IL1A, IL1B, NFKB complex, IL-1, IL-15, IL-17A, IL-18, Toll-Like Receptor (TLR)-4) and their upstream and downstream target molecules (Fig. 5b). Similar analysis of PD transfected cells revealed involvement of cell proliferation and growth related molecules such as GATA-4 and EZH-2. These results suggest that control siRNA, irrespective of the brand (Dharmacon or Invitrogen) can lead to activation of the immune and growth pathways at the transcriptional level.

### Validation of changes in key genes using qRT-PCR

**Fig. 6:**
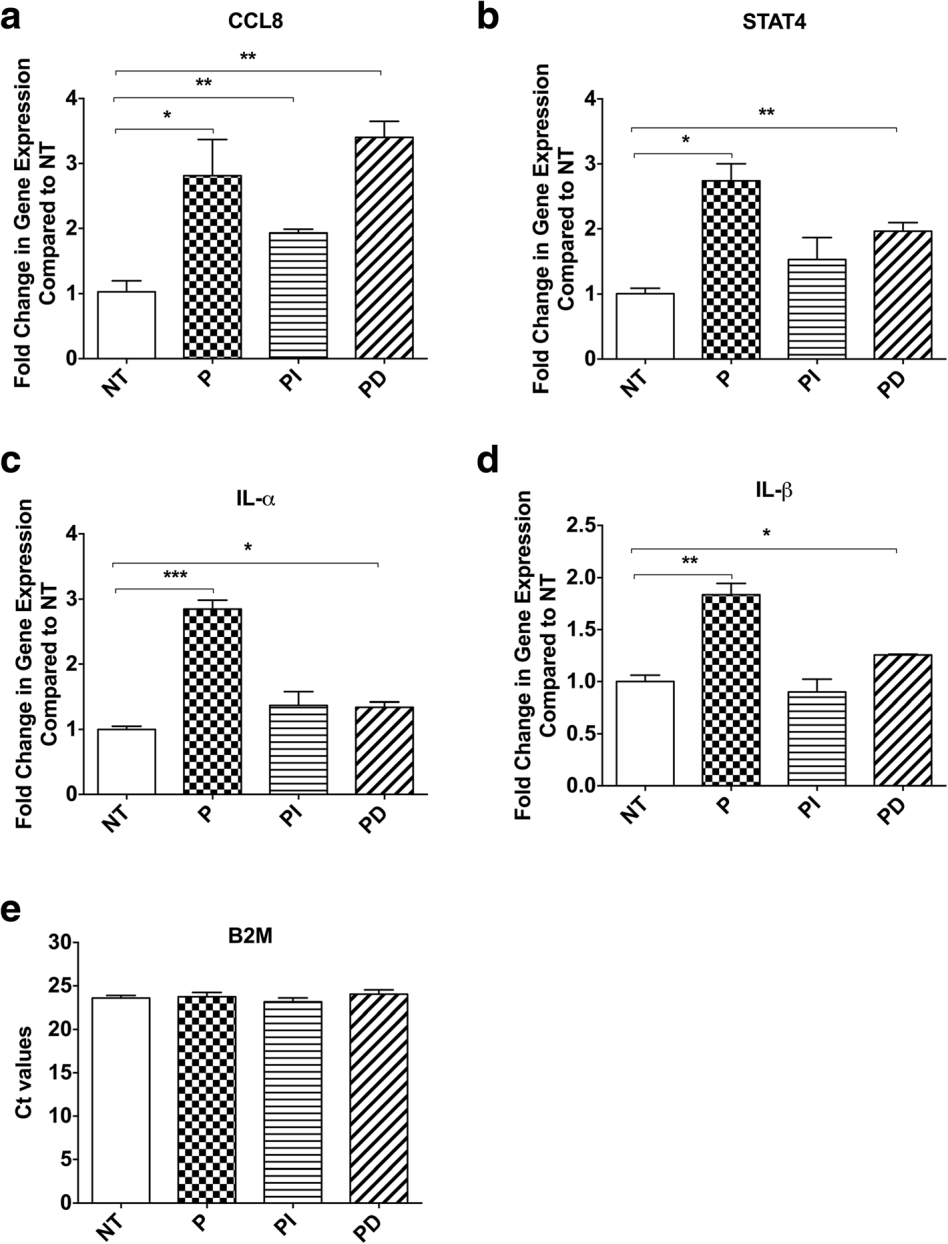
Validation of differentially regulated genes using qRT-PCR in HAoSMC that were either left untreated (NT) or treated with PEI alone (P), PEI combined with control siRNA from Invitrogen (PI), or Dharmacon (PD). Gene expression of (**a**) CCL8, (**b**) STAT4, (**c**) IL-1α, (**d**) IL-1β, and (**e**) raw Ct value of B2M. (*P < 0.05, **P < 0.01 and ***P < 0.001)

To confirm our findings from the RNASeq study using qRT-PCR we validated a few key genes that were differentially regulated and are known to be involved in vascular dysfunction [8–11]. Our results confirm that as compared to NT, HAoSMC treated with P, PI and PD show an upregulation of CCL8, STAT4, IL-1a and IL-1b gene expression (Fig. 6). Consistent with our RNASeq data, most significant effects were observed with HoASMC treatment with P and PD. With significant changes in global gene expression, housekeeping genes may also show differential expression, making them unreliable and thereby discrediting the qRT-PCR analysis. PEI is known to affect the gene expression of commonly used housekeeping gene Glyceraldehyde-3-Phosphate Dehydrogenase (GAPDH) [12], hence in this study we used another commonly used housekeeping gene, B2M. Expression of B2M gene was not different between the different treatment groups suggesting that B2M could be used reliably as a housekeeping gene.

## Discussion and Conclusions

We have examined in detail the transcriptome response to a commonly used transfection reagent, PEI alone or in combination with one of two commonly used control siRNAs (Invitrogen or Dharmacon). Our results demonstrate a broad and significant change in the transcriptome in response to the transcription reagent and a further change as a result of addition of the control siRNA.

Our data demonstrate that the transcription reagent PEI induces an extensive transcriptome response in cultured AOSMC. The affected genes include several genes that might be seen as targets in an RNAi therapeutic strategy such as for the treatment of vascular diseases, cancer or modifying the inflammatory response. For example, PTGS2 or Cylcooxygenase (COX)-2 that are altered in our present study are associated with atherosclerosis and have been shown to be upregulated by the prostacyclin-mimetic, iloprost in human vascular SMC [13]. Similarly, another altered gene in the study, NAMPT, is also linked to atherosclerosis and its inhibition has attenuated atherosclerotic plaque formation through CXCL-1 mediated activities on neutrophils [14]. The PEI effect is additionally altered when the control siRNA is combined with the PEI. Pathways analysis of commonly affected genes showed significant enrichment in inflammatory response pathways including Granulocyte/Agranulocyte Adhesion and Diapedesis. The genes involved in these pathways are the primary line of host defense against bacterial infection and are rapidly recruited to sites of bacterial invasion [6]. System biology analysis further demonstrated overexpression of inflammatory ctyokines such as IL-1, CCL-8, GATA-4 and EZH-2. IL1 and CCL8 are prominent inflammatory cytokines implicated in multiple inflammatory diseases such as atherosclerosis, rheumatoid arthritis (RA) and Alzheimer’s diseases [15, 16]. GATA 4 is involved in transcription and cell maturation and has been shown to be associated with multiple cancers and cardiovascular diseases [17–21]. EZH-2 activation results in increase in histone methylation causing inhibition of multiple tumor suppressor genes resulting in cancer progression. It is interesting to note that our results are contradictory to the findings obtained in the study of off-target effects in human fibroblast cells after being transfected with control siRNAs obtained commercially [22]. While present study showed enhancement in inflammatory responses, their study demonstrated inhibition of innate immune system through the reduction of NFκB signaling after the cells were transfected with control siRNAs [22]. However, it could be possible that this is a cell-specific effect. We have previously reported differential susceptibility of endothelial and smooth muscle cells to different transfection reagents [23, 24].

In the end, the transcriptome of a commonly used control for RNAi experiments is no longer representative of the transcriptome of untreated cells. Whether this response to PEI will hold for other transfection reagents remains to be seen. However until proven otherwise it is prudent to assume that is the case and not limit controls in RNAi cell culture studies to the transfection reagent combined with a control siRNA. The present study justifies the use of two additional controls: untreated cells, and transfection reagent alone. These controls will determine if the target gene expression is altered and whether it is a consequence of the transfection reagent alone or transfection reagent combined with a control siRNA.

Our data show that gene silencing studies using PEI in conjunction with either of the two popular control siRNAs must be regarded as relative to a very altered transcriptome. This places limits on the predictive value for clinical application of many gene-silencing experiments. For example, an experiment might be designed to see if siRNA can effectively diminish expression of IL-1, a key inflammatory cytokine involved in numerous diseases. Demonstration of effectiveness might then lead to consideration of therapeutic use of IL-1 siRNA to control the disease. However, the consequences of the transfection reagent and the control siRNA may increase expression of IL-1 to the point where, even with relative silencing, the expression of IL-1 is actually greater than in the resting cell state. It would then be a mistake to assume that the same RNAi strategy would be effective as a therapeutic in the control of the disease using IL-1 as a target. In fact, it might be harmful because the net effect would be an increase in expression of the undesired gene. Our data show the multiple biologic systems that respond to the transfection reagent, especially inflammatory and immune related pathways. This is understandable based on the cell membrane injury associated with PEI. Less clear is the source of the additional transcriptome response precipitated by the control siRNA.

With regard to therapeutic strategies, global expression consequences of a transfection reagent and non-coding siRNA might also be harmful by increasing expression of off target genes that have undesired effects. Or, the strategy might decrease expression of genes that have desired effects. Of note here, is that we have examined one time point, 24 h. However it is possible that dysregulated off target genes seen at 24 h might subsequently have downstream effects on the chosen target at a later time-point. These are important considerations in designing translational experiments using in vivo models. Before predicting the ultimate outcome of silencing a high profile target, it is necessary to follow the complete flare of the transcriptome response to the transfection reagent plus siRNA. This is extremely important for our work focused on the use on siRNA-based therapy for the prevention of graft failure. Vascular graft failure is a multi-factorial and sequential phenomenon with complex components including smooth muscle and endothelial cells. The work presented here is an attempt to understand the basic response of the smooth muscle cells to PEI and the different control siRNAs. Eventually, we will be conducting similar study in other cell types including endothelial cells before embarking on the use of PEI in an *in vivo* study. More importantly, irrespective of the disease model, any siRNA-based therapy will be subjected to a similar scrutiny before its use in an *in vivo* system.

Based on our study, it can be argued that the global transcriptome response of any RNAi based strategy should be determined before commencing clinical trials. Off target effects must be assumed and should be clearly defined to minimize the risk of doing more harm than good.

## Methods

### Cell culture

HAoSMCs (Lonza, Walkersville, MD) were cultured in basal medium Clonetics smooth muscle cell (SMC) medium (SmGM-2, CAT# CC-3181, Lonza) enriched with the supplied SMC growth additives (Insulin, CAT# CC-4021D; hFGF-B, CAT# CC-4068D; GA-1000, CAT# CC-4081D, FBS, CAT# CC-4102D, hEGF, CAT# CC-4230D, Lonza). The cells were maintained in a humidified incubator at 37 °C with 5 % CO_2_. Cells from passages 6–9 were used in the experiments.

### siRNA transfection

HAoSMCs were seeded at a density of 3x10^5^ cells/well in a 6-well flat-bottom plate. The total volume of each well was 3 ml. Twenty four hours later, cells were transfected with transfection reagent alone or with 50 nM of either control siRNA from Invitrogen (CAT# 4390843, Invitrogen, Carlsbad, CA) or control siRNA from Dharmacon (CAT#ID D-001206-13-20, Dharmacon, Lafayette, CO) according to manufacturer’s protocol. JetPEI™ (Polyplus, NY) with nitrogen in PEI to phosphate in siRNA (N/P) ratio of 10 was used as the transfection reagent. This ratio has been optimized in our recently published work [3]. Cells in culture medium without any treatment were used as experimental control. Master mix was created for each individual condition in order to eliminate pipetting errors and to increase consistency between each well. Each treatment condition was performed in triplicate.

### RNA isolation

After 24 h of transfection, HAoSMCs were lysed with Buffer RLT (Qiagen, Limburg, Netherlands). Total RNA was extracted and purified using RNeasy Mini Kit (Qiagen, Limburg, Netherlands) with an optional step: addition of DNase I mix to remove any genomic DNA contamination and to enhance the quality of RNA. RNA concentrations for each sample were obtained using NanoDrop 2000 spectrophotometer (Thermo Scientific, Wilmington, DE).

### Transcriptome profiling using RNA quantification sequencing

Transcriptomes derived from HAoSMCs that were untreated (NT), treated with PEI only (P), PEI with control siRNA from Invitrogen (PI) or PEI with control siRNA from Dharmacon (PD) were subjected to next-generation sequencing (NGS) to generate deep coverage RNASeq data. For each treatment group, sequencing was performed on three biological replicates. Sequencing libraries were generated from the double-stranded cDNA using the Illumina TruSeq kit according to the manufacturer’s protocol. Library quality control was checked using the Agilent DNA High Sensitivity Chip and qRT-PCR. High quality libraries were sequenced on an Illumina HiSeq 2000. To achieve comprehensive coverage for each sample, we generated ~25–30 million single end reads.

### RNASEQ data analysis

The raw RNA sequencing data was processed to remove any adaptor, PCR primers and low quality transcripts using FASTQC and Fastx softwares. These provide a very comprehensive estimate of sample quality on the basis of read quality, read length, GC content, uncalled bases, ratio of bases called, sequence duplication, adaptor and PCR primer contamination. Initially all Illumina TruSeq2 specific adaptors were removed. The bases from start and end of reads that are below quality threshold (Phred Score <20) were trimmed. The reads with average quality Phred quality score <20 were removed from further analysis. Post-trimming, the reads with length less than 36 were removed from analysis to avoid any non-specific mapping. The reads with >10 % N bases calls were removed from analysis. After preprocessing and filtering high quality, clean reads were aligned against human genome using tophat2 and bowtie2 packages (http://ccb.jhu.edu/software/tophat/index.shtml) [25]. We used hg19 human genome assembly as reference genome for alignment. Gene expression measurement was performed from aligned reads by counting the unique reads using htseq-count algorithm. The read count based gene expression data was normalized on the basis of library complexity and gene variation using the R package EdgeR [26]. The normalized count data was compared among groups using a negative binomial model to identify differentially expressed genes. The differentially expressed genes were identified on the basis of multiple-test corrected *P* value and fold change. Genes were considered significantly differentially expressed if the *p*-value was <5 % FDR and absolute fold change >2.

### Unsupervised analysis

Unsupervised analysis was performed using Principal Component Analysis (PCA), which projects multivariate data objects onto a lower dimensional space while retaining as much of the original variance as possible.

### Gene ontology (GO) analysis

To identify over-represented GO categories in differentially expressed genes, we used the Biological Processes and Molecular Functions Enrichment Analysis available from the Database for Annotation, Visualization and Integrated Discovery (DAVID) [27]. DAVID is an online implementation of the EASE software that produces a list of over-represented categories using jackknife iterative re-sampling of the two-tailed Fisher exact probabilities [27]. A *p*-value gets assigned to each category on the basis of enrichments. Smaller *p*-values reflect increasing confidence in over-representation. The GO categories with *p*-values <0.01 and at least 3 genes were considered significant.

### Pathway and interactive network analysis

Ingenuity Pathway Analysis (IPA 8.0, Qiagen) was used to identify the pathways and interaction networks that are significantly affected by significantly differentially expressed genes. The knowledge base of this software consists of functions, pathways and network models derived by systematically exploring the peer reviewed scientific literature. A detailed description of IPA analysis is available at the Ingenuity Systems’ web site (www.ingenuity.com). It calculates a *p*-value for each pathway according to the fit of users’ data to the IPA database using one-tailed Fisher exact test. The pathways with *p*-values <0.01 were considered significantly affected. For each network, IPA calculates a score derived from the *p*-value of one-tailed Fisher exact test [score = −log (*p*-value)] and indicates the likelihood of focus genes appearing together in the network due to random chance. A score of 2 or higher has at least a 99 % probability of not being generated by random chance alone. The ability to rank the networks based on their relevance to the queried data sets allows for prioritization of networks with the strongest association with pre-to post-intervention changes.

### Regulatory module analysis

The regulatory module analysis was used to identify the cascade of upstream transcriptional regulators that can explain the observed gene expression changes to help identify key regulators (master regulators) and understand the underlying biological mechanism [28]. The analysis helps in identifying first which transcription regulators are significantly affected by the treatment and then determine whether they are activated or inhibited. The activation or inhibition of transcriptional regulators was assessed by determining the overlap among users data with activation or inhibition signatures of regulators. The significance of overlap was determined using one tailed fisher Exact test.

### Quantitative real time PCR

Quantitative RT-PCR (qRT-PCR) was performed on each of the three biological replicates for each of the treatment conditions as described to validate changes in key genes [29]. Briefly, cDNA was obtained from 100 ng of total RNA using iScript reverse transcriptase from BioRad (Hercules, California). Specific gene targeting primers were obtained from Integrated DNA Technologies, (Coralville, Iowa). All primers were used at a concentration varying between 600nM and 900nM. Gene sequences are presented in Additional file 1: Table S1. PCR was performed using Brilliant III Ultra-Fast SYBR® QPCR Master Mix in conjunction with the Agilent Mx3005P PCR machine (Agilent Technologies, Santa Clara, CA). Beta-2-Microglobulin (B2M) was used as a house-keeping gene. For quantitative analysis, target gene levels were normalized to B2M levels as previously described, [30] and target and housekeeper gene amplification reactions were performed in duplicate for each of the cDNA sample (three biological replicate for each treatment condition) using 1 μl cDNA per reaction. For validation of differentially expressed genes, chemokine chemokine (C-C motif) ligand 8 (CCL-8), also known as monocyte chemoattractant protein 2 (MCP-2), Signal Transducer And Activator Of Transcription (STAT)-4, IL-1α and IL-1β, specific primers were used. All data are expressed as the mean ± SEM and are analyzed using One-Way ANOVA test. An unpaired *t*-test was performed and *p* < 0.05 was considered statistically significant.

### Supporting data

Additional information and data set(s) supporting the results of this article are included within the article as an Additional file 1.

## Additional file

Additional file 1:
**Supplementary Tables.** (XLS 138 kb)

## Abbreviations

B2M: Beta-2-microglobulin
CCL: Chemokine (C-C motif) ligand
CXCL: Chemokine (C-X-C motif) ligand
CSF: Colony stimulating factor
DAVID: Database for annotation, visualization and integrated discovery
EZH2: Enhancer of zeste homolog 2
FXR/RXR: Farnesoid X receptor/retinoid X receptor
GATA 4: GATA binding protein 4
GO: Gene-ontology
GAPDH: Glyceraldehyde-3-phosphate dehydrogenase
HAoSMC: Human aortic smooth muscle cell
IL: Interleukin
KIF: Kinesin family member
MAPK: Mitogen-activated protein kinase
MCP-2: Monocyte chemoattractant protein 2
NGS: Next generation sequencing
NAMPT: Nicotinamide phosphoribosyltransferase
N/P: Nitrogen in PEI to phosphate in siRNA
NFKB: Nuclear factor kappa beta
P: PEI only
PI: PEI with control siRNA from invitrogen
PD: PEI with control siRNA from dharmacon
PPAR: Peroxisome proliferator-activated receptor
PEI: Polyethyleneimine
PTGS: Prostaglandin-endoperoxide synthase
RNAi: RNA interference
STAT: Signal transducer and activator of transcription
siRNA: Small interfering RNA
SMC: Smooth muscle cell
SOD: Superoxide dismutase
TLR: Toll-like receptor
NT: Untreated
